# Functional measurement of canine muscular fitness: refinement and reliability of the Penn Vet Working Dog Center Sprint Test

**DOI:** 10.3389/fvets.2023.1217201

**Published:** 2023-07-03

**Authors:** Brian Farr, Jorie Gabrysiak, Rosemary Traylor, Sofia Zayas, Meghan Ramos, Amritha Mallikarjun, Cynthia Otto

**Affiliations:** ^1^Department of Defense Military Working Dog Veterinary Service, Joint Base San Antonio-Lackland, San Antonio, TX, United States; ^2^Penn Vet Working Dog Center, Clinical Sciences and Advanced Medicine, School of Veterinary Medicine, University of Pennsylvania, Philadelphia, PA, United States; ^3^Clinical Sciences and Advanced Medicine, School of Veterinary Medicine, University of Pennsylvania, Philadelphia, PA, United States

**Keywords:** dog, working, sporting, fitness, sprint, test, rehabilitation

## Abstract

Working, sporting, and companion dogs require muscular fitness to perform their daily tasks, competitive activities, and operational functions effectively and with a low risk of injury. There are currently no methods to measure the muscular fitness of dogs who are not debilitated. Sprint performance is highly correlated with muscular fitness in humans, and various sprint assessments are used to measure performance for sporting and tactical athletes. The Penn Vet Working Dog Center Sprint Test (ST) is a 25 m maximal effort sprint from a down position and was developed to be a low-cost measure of muscular fitness suitable for field use. The purpose of this project was to describe the refinements to the ST, detail the performance and measurement protocol, evaluate the measurement inter-rater and intra-rater reliability, characterize the acceleration profile, and explore the inter-day reliability. Both naïve and experienced raters demonstrated excellent intra-rater and inter-rater reliability. The acceleration profile of the dogs in this study was similar to that of average adult human sprinters and demonstrated the role of muscular fitness in performance over this short distance. Finally, a small group of dogs showed moderate inter-day reliability and provided initial performance data to inform future studies. The ST appears to be a reliable measure of canine muscular fitness and could be used to assess performance in healthy dogs and guide the return to sport or work of debilitated dogs.

## Introduction

All dogs should be physically fit enough to perform their daily activities and those supporting their relationships with other dogs and people (e.g., walks and playing). Some dogs perform more physically demanding tasks while training for or competing in sports or while training for or performing operational tasks like detection or apprehension. Dogs lacking the physical fitness required for their tasks risk diminishing their ability to perform them or becoming injured while performing them. Compared to pet dogs, fitness is of even greater importance for working dogs as insufficient fitness may result in failing a task and subsequent injury to their human (and animal) teammates or another human (1–3).

Companion dogs, sporting dogs, and working dogs need well-rounded fitness, including the modalities of strength, power, mobility, cardiorespiratory endurance, balance, muscular endurance, and agility (4). The three elements of muscular fitness are strength, muscular endurance, and power, and these are defined as “the ability of muscle to exert force,” “the ability of muscle to continue to perform without fatigue,” and “the ability or rate at which one can perform work,” respectively (5). Dogs need muscular fitness to walk and trot, sprint, slow down and stop, jump up and down, navigate obstacles and uneven surfaces, and stand on their hindlimbs. While the evidence for the role of muscular fitness in dogs is predominantly limited to rehabilitation from injury, disease, or age-related decline (6–8), there is extensive human research on the benefits of muscular fitness for health, injury risk reduction, competitive performance, and task performance (9–11).

Attaining the necessary level of muscular fitness involves determining the current level through assessment, pursuing an appropriate training program, reassessing periodically, and repeating these elements until the required level is reached. For optimal utility, an assessment should be reliable (measurement and test–retest), use a minimum of equipment, and be simple to perform (12). Muscular fitness in dogs is primarily assessed through daily task performance and muscle mass measurement (13–15), and other performance-based assessments have been proposed (16–18). Several canine sporting events (e.g., Fast CAT and Dock Diving) are highly standardized performance assessments that require significant contributions from muscular fitness. Muscular fitness is assessed in numerous ways in humans, and some competitive events are direct assessments of these modalities (e.g., track and field). Performance on short-distance sprinting, for example, is highly correlated with measures of power (19), strength (20), muscular endurance (21), and with trunk muscle mass (22). Off the athletic field, muscular fitness is also critical for human tactical athletes, those personnel in law enforcement, the military, or rescue services (23–26). Assessment of muscular fitness for these athletes is common, with the Army Combat Fitness Test including assessments of strength (3 Repetition Maximum Deadlift), power (Standing Power Throw), and muscular endurance (Hand-Release Push-Up, Sprint-Drag-Carry, and Plank) (27).

A gap exists in determining the muscular fitness of dogs whose abilities exceed the capacity of current assessments. An objective, simple, muscular fitness assessment directed at high-performance dogs would aid in guiding dogs in the return-to-work or sport phases of rehabilitation and inform operational decisions like deployment and retirement (28). The Penn Vet Working Dog Center (PVWDC) Sprint Test (ST) was proposed as a method to fill this gap (4). The ST is as an assessment of a dog’s ability to generate whole-body power during the acceleration phase (first 25 m) of sprinting. The propulsion for sprinting involves forelimb (shoulder flexion, elbow extension, and carpal flexion), trunk (spinal flexion, extension, and stabilization), and hindlimb (hip, stifle, and tarsal extension) components with the hindlimb supplying the majority (29–32). The ST uses video timing to estimate the duration to complete the distance, and this timing method has been shown to be as accurate as the fully automatic photo finish systems used in international human competition (33).

There were several purposes for this study. The first purpose was to describe in detail the ST setup and execution protocol that were introduced in our original methods paper (4). The next purpose was to develop a training program for novice evaluators and assess the measurement reliability for evaluators with a range of experience levels. We hypothesized that a suitable training program could be developed to quickly enable a novice to measure ST performance with nearly the same accuracy and precision as an experienced evaluator. The third purpose was to characterize the acceleration profile of a dog performing the ST and compare it to data from human athletes. We hypothesized that these would be similar. The final purpose was to evaluate the inter-day reliability and describe the performance of a small group of working dogs. We hypothesized that the ST would have a small amount of inter-day variation, and this population would show the expected biological variation in performance.

## Materials and methods

### Animals

All canine participants were either working dogs in training or working dogs performing detection research at the PVWDC (Philadelphia, PA, United States). Dogs conducted their normal training in addition to the study. On musculoskeletal examination by one of the study veterinarians, all dogs were considered sound.

To be included the dogs had to be one of the typical working breeds, over 0.67 years of age, free of any performance-limiting condition, and familiar with the ST process. Dogs were excluded from the study if they were under 0.67 years old, had a performance-limiting issue, were not present for the entire study, and/or were not familiar with the ST process.

### ST setup and performance

**Figure 1:**
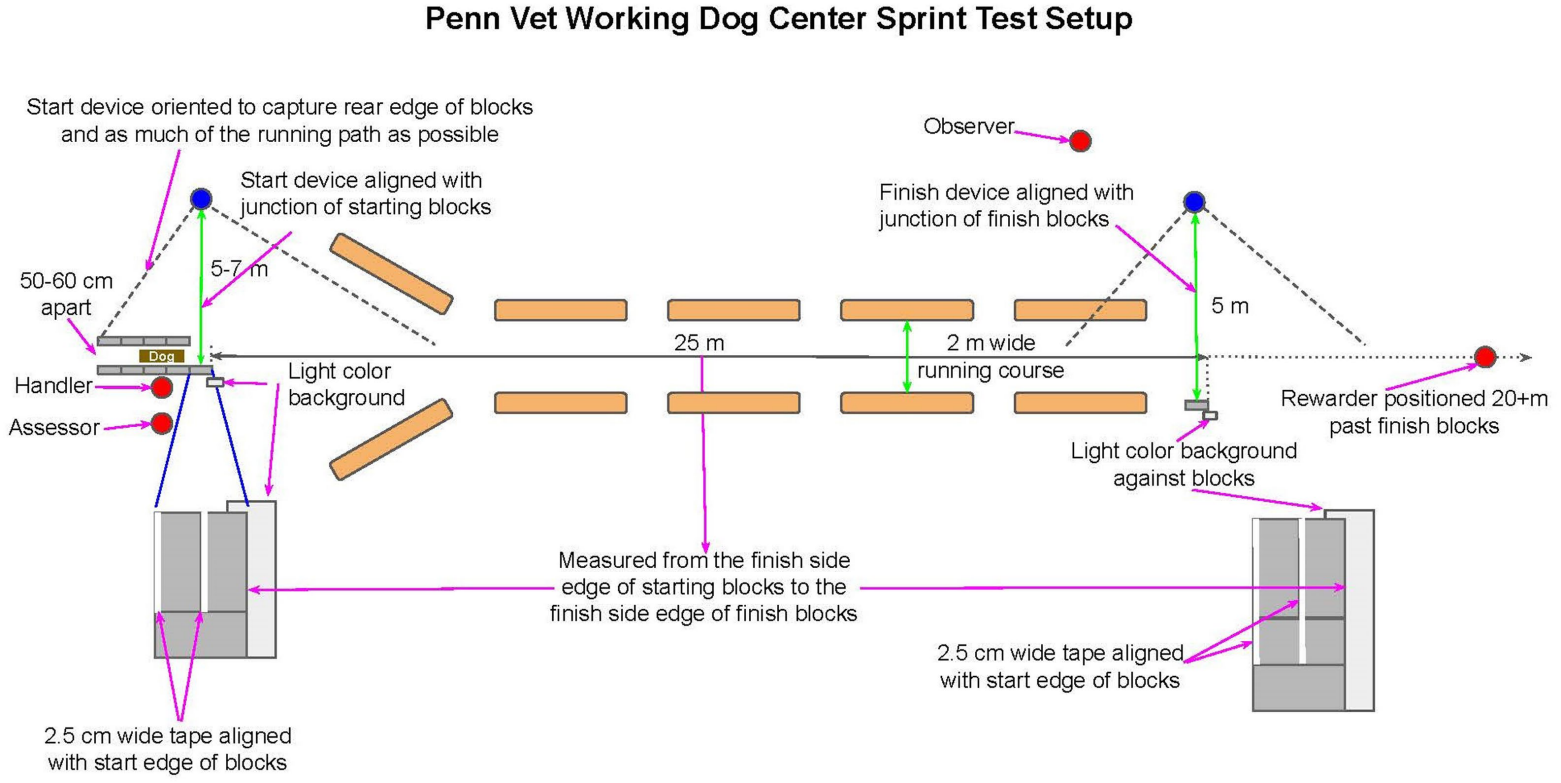
Overview of the ST setup.

**Figure 2:**
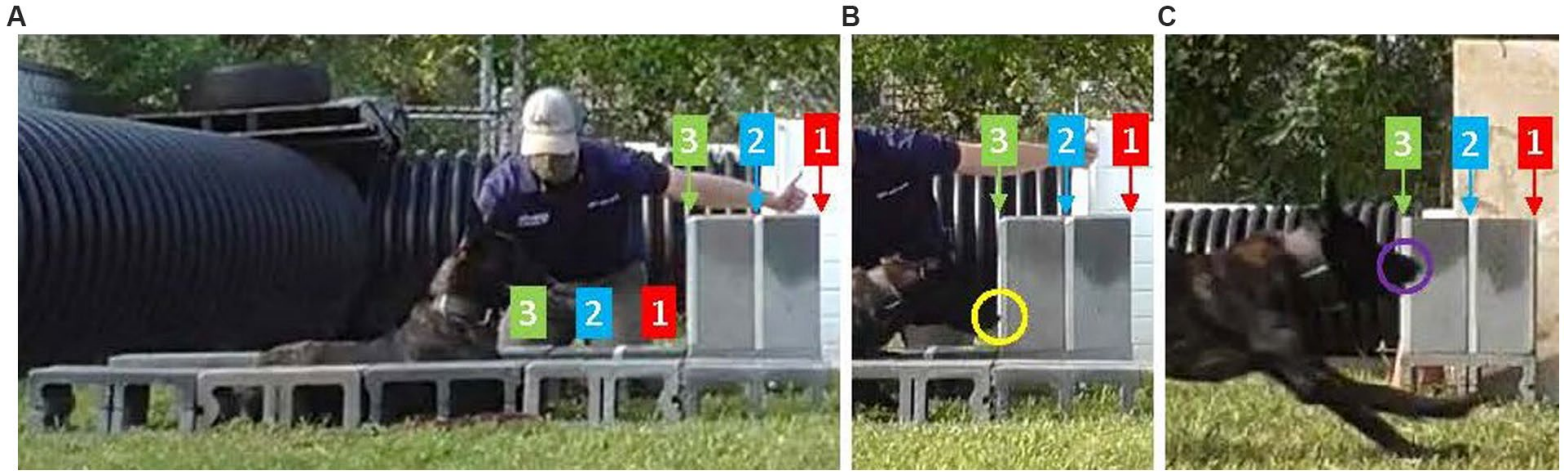
**(A)** Shows the ST start setup with the dog’s chest in Zone 3. **(B)** Shows the dog’s nose crossing start line 3 (yellow circle). **(C)** Shows the finish setup with the dog’s nose crossing finish line 3 (purple circle).

The ST setup and performance procedure is described in detail in the Supplementary material and summarized in Figures 1, 2. Briefly, the ST is a 25 m sprint from which the dog starts in a stationary down position. A highly motivating reward is used to incentivize maximal performance. Three starting zones corresponding with three start and finish lines are used to accommodate the dog’s behavior (weight shifting or small movements forward) when highly aroused. The start and finish are captured on video [at least 60 frames per second (fps)] using recording devices synchronized by viewing the light from a flashlight. The dog performs 3–5 attempts, and the performance on the fastest attempt is designated as the test result. The dog’s perceived effort [graded on a four-tier declining scale from highly motivated, “A,” through decreasing visible effort (“B” and “C”) to failed to complete, “F”] and test quality [graded on a four-tier declining scale from no issues “A,” through increasing issues (“B” and “C”), to unable to complete “F”] are observed and reported.

### ST measurement

The ST measurement procedure is described in detail in the Supplementary material and summarized in Figure 2. Briefly, the start and finish videos are analyzed to determine the dog’s quantitative and qualitative performance. This study used Kinovea (version 0.8.27), a free 2D motion analysis software running on desktop computers to perform frame-by-frame analysis. Both videos were analyzed to determine the frame when the synchronizing method was first visible. The start and finish videos were analyzed to determine the start and finish times of each attempt. Any abnormalities in the dog’s movement or unrecognized perceived effort or test quality issues were noted. The duration of each attempt was calculated by adjusting the start time (Ts) and finish (Tf) times by the synchronize time (STs and STf) and then subtracting the adjusted start time from the adjusted finish time [e.g., (Tf-STf) – (Ts-STs)]. The shortest duration from a properly performed attempt (“A” for both perceived effort and test quality) was then reported as that dog’s result.

### Measurement reliability

Four raters were recruited for measurement reliability. One rater was to be highly experienced with measuring the ST (had rated more than 200 dogs performing the ST), one rater was to be moderately experienced (had rated 25–100 dogs performing the ST), and two raters were to have no prior experience measuring the ST before the training procedure.

#### Training procedure

The highly and moderately experienced raters measured the start and finish videos of five previously recorded dogs performing three attempts each. The highly and moderately experienced raters’ measurements for the STs, STf, and the Ts and Tf from each attempt for each dog were compared. The two raters discussed any differences and agreed on the actual measurement that would serve as the standard for the novice raters.

The novice raters were provided a standardized measurement protocol and a demonstration video to familiarize themselves with Kinovea. The novice raters reviewed the protocol and demonstration video and then discussed any questions with the highly experienced rater. After becoming familiarized with the process, the novice raters measured a training set of videos. They were provided with the start and finish videos of the same five dogs as above, and for efficiency of review, a spreadsheet identifying the approximate start and finish times of each attempt (rounded down to the nearest second) and the starting zone for each of the three attempts for each dog. Both novice raters measured each video to identify the STs, STf, Ts, and Tf for each attempt. These times were recorded to the millisecond, entered into an online form, and submitted. Both novice raters then measured the five dogs’ videos again in a different order after an interval of 3 days.

The initial and repeat results of each novice rater were then compared to each other and to the measurement standard set by the highly and moderately experienced raters. The novice raters were provided feedback and provided the opportunity to remeasure any incorrect measurements. All raters were considered trained when 80% of their measurements exactly matched the measurement standard, and any measurements that did not match differed by only one frame in either direction.

#### Measurement reliability procedure

All 4 raters were provided with the start and finish videos of 23 dogs performing a total of 83 attempts (3–5 per dog) and a spreadsheet with the information for each attempt like that provided during the training procedure. Each rater measured all the attempts in each video and submitted their results using an online form. Three of the raters then measured all the attempts in each video again in a different order after an interval of 7 days.

The inter-rater and intra-rater differences for all measurements (STs, STf, Ts and Tf for each attempt, and the calculated duration for each attempt) were assessed. Due to the small variation in each of these individual measurements, the calculated duration for each attempt was selected for further analysis. This analysis was conducted in R (version 4.1.2) (34), and intraclass correlation coefficients (ICC) were determined using the IRR package (version 0.84.1) (35) based on a single observation, consistency, one-way model. The inter-rater reliability was determined by calculating the ICC for the first measurement attempt for all raters. The intra-rater reliability was determined by calculating the ICC for the initial and repeat measurement for each rater. The actual inter-rater difference was determined by first averaging the duration for each rater’s first measurement to create a reference standard. Next, the absolute difference between this value and the duration for each rater’s first measurement was determined. Finally, this difference for all raters was averaged for each attempt, and the overall mean and standard deviation were determined.

### Acceleration profile characterization

Two dogs were recruited for this portion of the study. All ST attempts were performed in one session in the same location, and the dogs were positioned to start from the first starting zone. Additional identical video cameras and narrow, vertical, and high contrast marking lines were placed at 5 m intervals along the course at 5, 10, 15, and 20 m. All video cameras were synchronized, and each dog performed attempts until three attempts with both “A” perceived effort and test quality scores were obtained. The resulting attempts were measured using just the start and finish cameras by a single rater (BF) to determine the attempt with the shortest duration. All videos obtained for this attempt were then reviewed and the dog’s progress was measured to determine the time to reach each marker. The duration between markers, average velocity (v) for each 5 m split, maximum velocity (v_max_), and percent of maximum velocity (%v_max_) were then calculated.

### Inter-day reliability

Eight dogs were recruited for this portion of the study. Two ST iterations were performed 1 week apart on the morning of the first training day of the week prior to any other training. No attempt was made to standardize or restrict the activities on the days preceding this first training day. Both iterations took place at the same location and involved the same personnel filling the same roles for each dog. Each dog performed attempts until three attempts with both “A” perceived effort and test quality scores were obtained. The resulting videos were measured by a single experienced rater to determine the attempt with the shortest duration from each iteration. To ensure measurement reliability, the videos of two of the eight dogs from each iteration were randomly selected for measurement by another rater. The results of the two raters were analyzed and compared to the inter-rater reliability data obtained during the measurement reliability procedure. The shortest durations from both iterations for each dog were compared, and the relative (ICC) and absolute (coefficient of variation (CV)) reliability were calculated. The ICC analysis was run in R using the IRR package based on a single observation, agreement, two-way mixed effects model.

## Results

### Refinement

The revised setup and execution protocol leveraged nearly 2 years of utilization, hundreds of canine athlete testing sessions, and thousands of attempts to bring needed clarity to the ST. The original description of the ST utilized a single camera at the finish to identify the first motion of the dog. This method was determined to be inaccurate due to the challenges with identifying small motions from over 25 m away. Early unpublished research to develop measurement training using this method showed inter-rater and intra-rater actual differences of approximately 0.1 s each. Refinement was needed to measure the ST more precisely and accurately.

The first refinement was adding a second camera at the start to better visualize the dog’s initial movement. A simple method to quickly synchronize the two cameras was needed, and the shared light flash was introduced. This second camera allowed greater visibility of the dog’s movements before and during the start, and a method to accommodate these movements and more precisely measure the start had to be developed. In the original version, dogs did not always start oriented parallel to the running path. The concrete block channel structured the dog’s position, and the starting zone concept associated with these blocks accommodated the inevitable movement of motivated dogs better than a single line. When viewed at 60 fps, dogs showed several starting movement patterns. Some moved their forefoot first, some dropped their head first, and some moved their entire body as a unit in a forward and upward motion. The starting point was therefore shifted from the “first motion,” and the nose was selected to be a readily identified marker crossing a newly developed starting line. This line had to be close enough to the dog to still capture much of the initial movement off the ground while also being far enough away to not be already crossed by a dolichocephalic dog positioned at the front of the zone and leaning forward. The size of the concrete blocks assisted in this process, and the starting line is generally 20–40 cm away from the dog’s nose when they first begin moving. This distance is covered in 5–10 frames and enables clear identification of the starting point while minimizing the lost portion of the dog’s initial movement.

The ST was originally developed for young working dogs in training, and their behavior during early testing iterations mandated several further refinements of the protocol. Many dogs were challenged to perform a down position in a specific 20 cm zone while anticipating a sprint toward their favorite reward. Two additional zones and corresponding starting and finish lines were added, giving dogs more flexibility to perform the task successfully. The visual and physical barriers were added along the running path to reduce distraction and encourage straight movement. Dogs showed varying levels of motivation during the ST, and human error was also found to affect the dogs’ performance. The perceived effort and test quality metrics were developed so that this qualitative information could be observed, scored, and recorded to add context to dogs’ quantitative performance.

### Measurement reliability

The highly experienced evaluator was a veterinary researcher who developed the measurement method in this project and had previously measured approximately 250 dogs performing approximately 1,000 attempts. The moderately experienced evaluator was an undergraduate research intern who had previously measured approximately 50 dogs performing approximately 200 attempts. This evaluator did not measure the videos a second time, and her results were therefore only included in the inter-rater evaluation. The two novice evaluators had observed the ST being performed but had no experience measuring the videos prior to the training procedure.

The novice raters reached the training standard after one round of remeasuring the videos (approximately 3–5 total attempts) they measured incorrectly during the first round. The inter-rater ICC for all raters was 0.995 (excellent) with a measurement duration difference of 0.014 (+/− 0.008) seconds (or 1–2 frames). The intra-rater ICC for the highly experienced rater was 0.997 (excellent) with a measurement duration difference of 0.015 (+/− 0.017) seconds. The intra-rater ICCs for the two novice raters were 0.997 and 0.996 (both excellent) with measurement duration differences of 0.018 (+/− 0.015) seconds and 0.022 (+/− 0.019) seconds, respectively.

### Acceleration profile characterization

**Figure 3:**
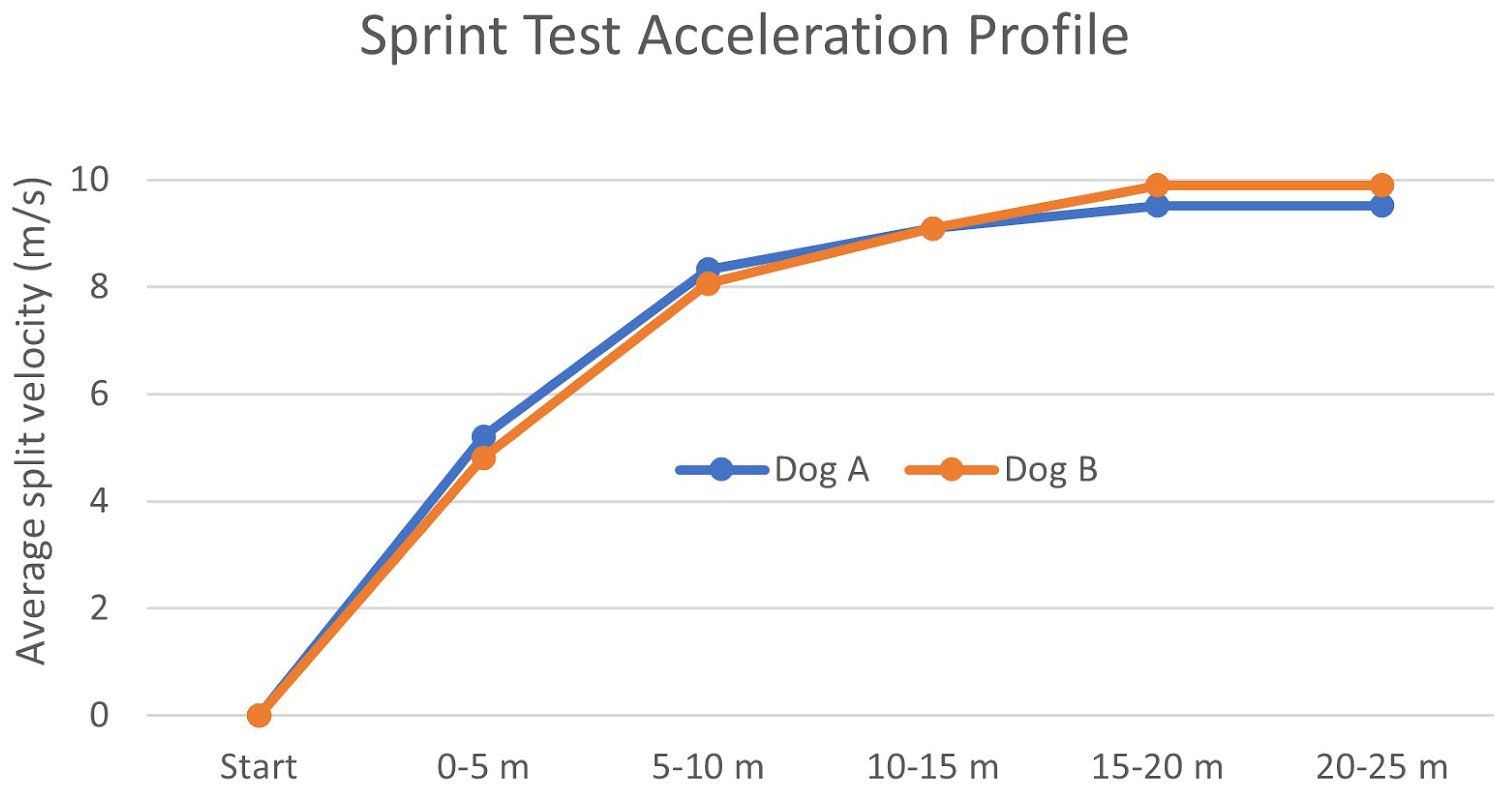
Acceleration profile of the average split velocity for each dog’s fastest attempt during 5 m segments of a 25 m sprint.

Two Labrador Retrievers participated in this portion of the study. Dog A was a 0.8-year-old intact male that weighed 27.9 kg, had a body condition score (BCS) of 3/9, was 61 cm tall at the withers, and was in training to be a search and rescue working dog. Dog B was a 3.0-year-old spayed female that weighed 21.6 kg, had a BCS of 6/9, was 55 cm tall at the withers, and was performing detection research. Both dogs achieved their v_max_ during the 15–20 m split and maintained that velocity during the 20–25 m split. The acceleration profile for both dogs is summarized in Table 1 and Figure 3.

**Table 1:** Average split velocity (v), split velocity relative to maximal velocity (%v_max_), overall time, and overall average velocity (v) for each dog’s fastest attempt.

	0–5 m		5–10 m		10–15 m		15–20 m		20–25 m		0–25 m	
	v (m/s)	%v_max_	v (m/s)	%v_max_	v (m/s)	%v_max_	v (m/s)	%v_max_	v (m/s)	%v_max_	Time (s)	v (m/s)
Dog A	5.21	54.7%	8.33	87.5%	9.09	95.5%	9.52	100.0%	9.52	100.0%	3.16	7.91
Dog B	4.81	48.6%	8.06	81.5%	9.09	91.8%	9.90	100.0%	9.90	100.0%	3.22	7.76

### Inter-day reliability

Three German Shepherd Dogs, three Labrador Retrievers, one Dutch Shepherd, and one Small Münsterlӓnder participated in this portion of the study. Five dogs were in training for working careers (dual purpose patrol and detection law enforcement, single purpose detection law enforcement, or urban search and rescue), and three dogs were performing detection research. All dogs were intact, five were male, and three were female. The median age was 2.2 years (range 0.8–5.7), the weight was 29.0 ± 5.0 kg, the BCS was 4.3 ± 0.9 on a 9-point scale, and the height at the withers was 62.3 ± 4.8 cm. The shortest duration of each dog’s best attempt across both iterations was 3.06 ± 0.098 s. The inter-day reliability ICC was 0.62 (moderate) with a CV of 3.2%.

## Discussion

All dogs should be fit enough to perform their life, sport, and/or work tasks, and muscular fitness is an important aspect of fitness. Sporting and working dogs require a higher level of muscular fitness, and the PVWDC ST has been proposed as a method to assess this fitness in a simple manner suitable for the temperaments common to these dogs (4). In this study, we described the protocol to set up, perform, and measure the refined ST. We demonstrated the excellent intra-rater and inter-rater measurement reliability possible using this method, explored the acceleration profile of the ST, and captured the performance and inter-day reliability of a small group of working dogs.

The measurement reliability portion of this study showed the refined ST could be measured with excellent measurement reliability and actual inter-rater and intra-rater differences of 0.02 s or 1–2 frames at 60 fps. These results were obtained by naive raters after undergoing a short, standardized training protocol. Like other video measurement studies, the ST can be measured to the limit of the frame rate of the recording device (33). This precision is critical when identifying small changes in performance and muscular fitness.

We developed the ST as a measure of muscular fitness and aimed to capture most of the initial acceleration without the influence of top speed or speed endurance. A group of average human sprinters achieved 96.2% of their maximum velocity in the 10–20 m split and 97.1% in the 20–30 m split (36). The current male 100 m world record holder, Usain Bolt, achieved 80.4% of his maximum velocity in the 10–20 m split and 90.2% in the 20–30 m split of his 2008 Beijing Olympic Games performance (37). When developing the ST, we selected the 20–30 m distance and created the 25 m distance to balance the capture of acceleration performance with a distance likely to fit (with additional space required before and after this distance) within the limitations of existing fenced training areas. Various distances have been used in human athletics to evaluate sprint performance and correlate that performance with other fitness and performance metrics (38–41). The current ST is a first step toward that goal for canine athletes.

To our knowledge, the acceleration profile of sprinting dogs has not been previously characterized. The acceleration profile of the two dogs in this portion of the study was more like that of average adult human sprinters than the current world record holder (36, 37). These dogs reached their maximum velocity in the 15–20 m split and then maintained this velocity through the 20–25 m split.

A small group of mature working dogs provided initial information on the ST. The inter-day reliability for this population in this study was 0.62 (ICC) and 3.2% (CV). To our knowledge, the inter-day reliability of canine sprint performance has not been reported. The accepted standard for similar distances in human athletics is ICC > 0.75 and CV < 3.0% (39). The causes of the lower inter-day reliability of the dogs in this study are not known. We hypothesize that variations in physical activity in the days preceding the testing days (over the weekend) and subtle alterations in test personnel behavior (especially the rewarder) may have contributed. The performance of the dogs in this study was 3.06 ± 0.098 s, and this information can assist future research and inform the development of performance standards.

The primary strength of this project is the description of a method to assess canine muscular fitness in the field environment. To our knowledge, the ST is the first standardized measure of sprint performance and muscular fitness in canine athletes. The measurement reliability portion showed that naive raters could be trained to effectively measure the ST. Finally, the inter-day portion of this project provided pilot data on performance and inter-day reliability to inform future research. This project can serve as the launchpad for future measurement of sprint performance and correlation with sporting and working ability (42).

The limitations of this project involved the number and type of dogs involved in the inter-day reliability portion. The dogs participating in this portion of the study were a convenience sample of lean medium and large breed working dogs in training and dogs performing detection research. Thus, their best performance and inter-day reliability may not be reflective of other canine athlete populations. The ST methodology should be evaluated in dogs of varying sizes and temperaments. We did not compare ST performance with other measures of muscular fitness. Future studies could address these limitations by conducting the ST on large groups of working dogs performing similar tasks (e.g., military working dogs or dual purpose law enforcement dogs) and by comparing ST performance with other measures of muscular fitness, including thigh circumference, thigh circumference relative to limb length, and measurements of muscularity using imaging techniques.

The ST appears to be an effective way to assess the muscular fitness of a canine athlete in a field setting. The ST could be used before and after a muscular fitness training program to determine the effect of the program or to compare the effects of different programs. Performing the ST on a large group of similar dogs could illuminate potential effects of age and career or sport duration on performance and assist in establishing population-specific standards. The ST could be used serially during the return-to-sport or work phase of rehabilitation to stage recovery and inform utilization, competition, or retirement decisions.

## Data availability statement

The raw data supporting the conclusions of this article will be made available by the authors, without undue reservation.

## Ethics statement

The animal study was reviewed and approved by the Institutional Review Board of the University of Pennsylvania Institutional Animal Care and Use Committee.

## Author contributions

BF, MR, and CO contributed to the conception and design of the study. BF, JG, RT, and SZ collected the data. BF and AM performed the statistical analysis. BF wrote the first draft of the manuscript. All authors contributed to manuscript revision, read, and approved the submitted version.

## Conflict of interest

The authors declare that the research was conducted in the absence of any commercial or financial relationships that could be construed as a potential conflict of interest.

## Publisher’s note

All claims expressed in this article are solely those of the authors and do not necessarily represent those of their affiliated organizations, or those of the publisher, the editors and the reviewers. Any product that may be evaluated in this article, or claim that may be made by its manufacturer, is not guaranteed or endorsed by the publisher.
