# Supplementary material for: Functional measurement of canine muscular fitness: refinement and reliability of the Penn Vet Working Dog Center Sprint Test
Source: Front Vet Sci. 2023 Jul 3;10:1217201. doi: 10.3389/fvets.2023.1217201 (PMC10350527; doi:10.3389/fvets.2023.1217201)
Supplement: Supplementary file 1 [file Data_Sheet_1.pdf]

Supplementary Material

Functional Measurement of Canine Muscular Fitness: Refinement and Reliability of the Penn Vet Working Dog Center Sprint Test

Brian Farr*, Jorie Gabrysiak, Rosemary Traylor, Sofia Zayas, Meghan Ramos, Amritha Mallikarjun, Cynthia Otto

*** Correspondence:** Brian Farr: brian.farr.dvm@gmail.com

1 Setup Protocol

1.1 Location

The Penn Vet Working Dog Center (PVWDC) Sprint Test (ST) is performed in an enclosed (fenced) area with minimal distractions. Either indoor or outdoor areas are suitable, but performing the ST outdoors may expose the dog to heat and humidity and slippery surfaces from rain or frost. The ST must be conducted on a high-traction surface suitable for sprinting, such as grass, dirt, or artificial turf. The surface must be level without holes or hazards like rocks, weeds, or tree roots. Any training equipment in the area must be situated to limit distraction for the dog and injury for the people performing the test.

The ST requires an area at least 10 m wide (2-4 m running course and 1-2 m on each side for barriers) and 40 m long (5 m starting area, 25 m course, and at least 10 m for rewarding past the finish line) with a length of 50-75 m being preferred to limit slowing down before the finish and facilitate safe rewarding away from obstacles. The ST setup is summarized in **Figure 1**. For this study, the ST was performed in an outdoor fenced grass area that was 30 m wide and 40 m long. The area was typically used for agility training, and the equipment was moved to accommodate the ST.

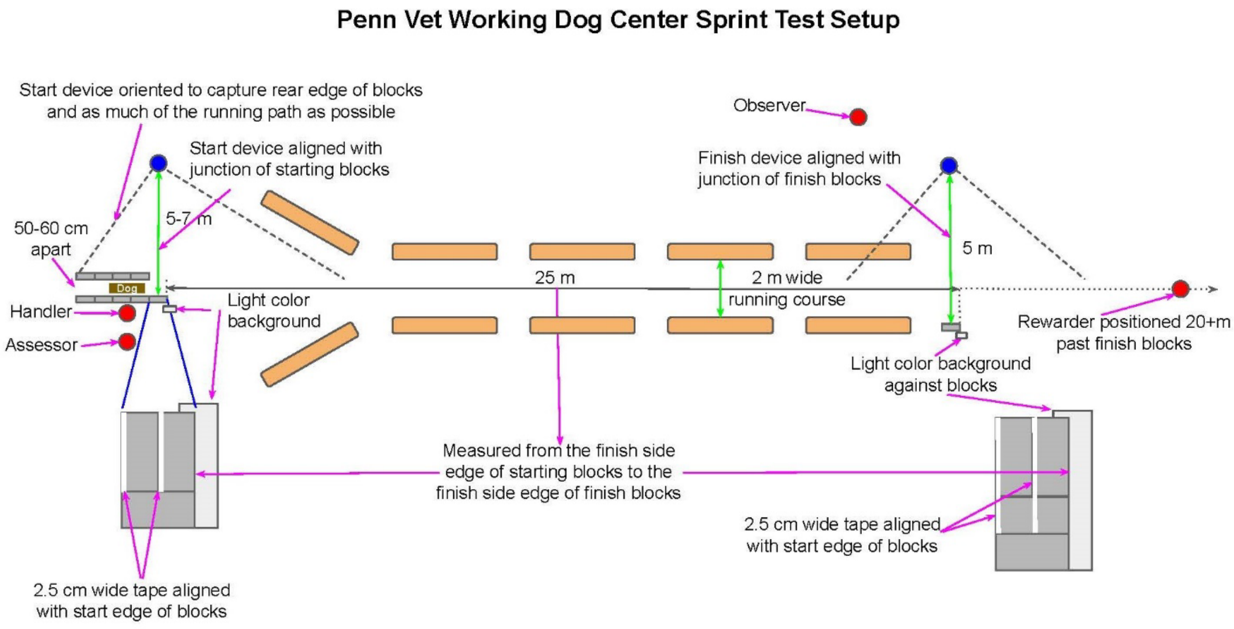

FIGURE 1**.** Overview of the ST setup.

1.2 Personnel Requirements

The ST requires a minimum of two people to perform, but three are preferred, and four may be required depending on the dog's temperament. One person is positioned at the start, handles the dog for the start (if the dog's temperament allows for it), observes the dog's starting position, signals their readiness to the second person, and observes and comments on the quality of the start. The second person is positioned near the finish, motivates the dog to sprint, and rewards the dog at the end of the sprint. The third person, if available, is positioned to the side of the course near the finish to observe and comment on the quality of the entire attempt. A fourth person may be needed if the dog's temperament does not allow the first person to handle them (e.g., the first person is not that dog's assigned handler). In this case, the fourth person handles the dog at the start while the first person performs the rest of their original roles.

1.3 Directional Terms

Four terms are used to describe directions relative to the running course and recording devices. The two ends of the short axis of the running course are referred to as the "start side" (the "near" side at the beginning of the running course) and the "finish side" (the "far" side at the end of the running course). The two long axis sides of the running course are referred to relative to the start and finish recording devices (e.g., "side closest to" and "side away from").

1.4 Start Setup

The ST start setup enables the dog to be oriented properly towards the finish, their relative starting position to be observed, and a smooth initiation of the attempt. The ST start setup is shown in **Figure 2 (A)**. Two parallel rows of standard 20 cm x 20 cm x 40 cm (8 in x 8 in x 16 in) concrete blocks are positioned behind the starting line to orient the dog towards the start. Five blocks are arranged in a

line with their short sides touching, and this line is placed on the side away from the start recording device. The first block of this line is assessed with a level to ensure it is level, primarily in the axis parallel to the running course.

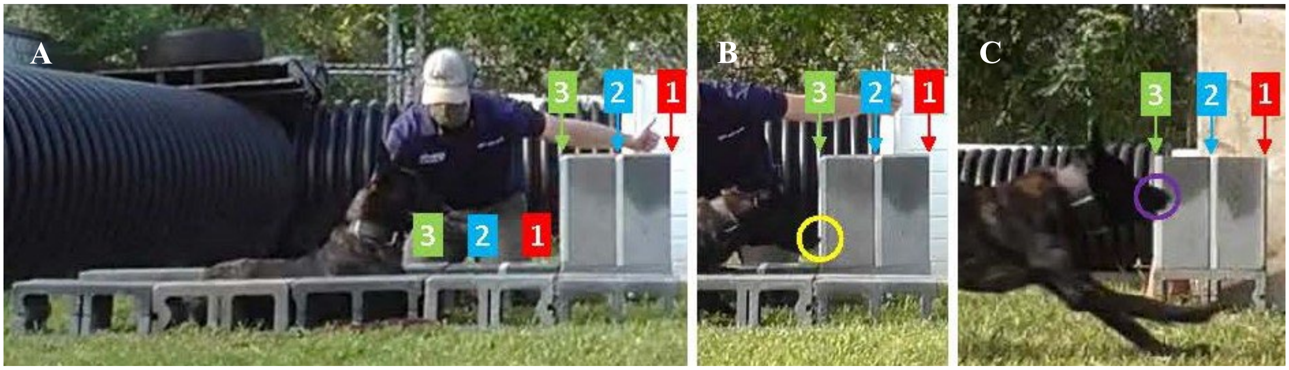

FIGURE 2. (A) Shows the ST start setup with the dog's chest in Zone 3. (B) Shows the dog's nose crossing Start Line 3 (yellow circle). (C) Shows the finish setup with the dog's nose crossing Finish Line 3 (purple circle).

Four blocks are arranged in a similar line, parallel to the first line, and on the side nearest the start recording device. This line does not include the block closest to the starting lines to enable visualization of the dog's nose as the head often dips down at the start. If the blocks have holes through them, the solid sides are oriented towards the top and bottom to reduce the risk of injury from a dog stepping into the holes. The lines of blocks are 30-50 cm (12-20 in) apart with the spacing being narrower for smaller dogs and wider for larger dogs or those apprehensive to move into a narrow space.

Two more blocks are stacked on top of the first block of the line away from the start recording device with their long edges together (oriented vertically). These blocks form the three starting lines. The first is formed by the finish side edge of the first block, the second by the junction between the two blocks, and the third by the start side edge of the second block. If the blocks have holes through them, the solid sides are oriented towards the start recording device. As most blocks are slightly (e.g., 0.25 in or 0.5 cm) shorter in height than half of their length, the two vertical blocks are centered (in the axis parallel to the running course) on the horizontal block below them. The vertical edges of these blocks are assessed with a level to ensure they are vertical, primarily in the axis parallel to the running course.

A strip of 2.5 cm (1 in) wide light-colored (e.g., white or off-white) tape (e.g., masking tape) is adhered to the long edges of the two vertical blocks away from the finish. The two tape strips are continued onto the horizontal block below. These strips provide clear identification when a dog's nose has crossed the start side edges of the first and second blocks (Starting Line 2 and Starting Line 3 respectively). A high-contrast (e.g., white or off-white) object is placed behind the edge toward the finish of the first vertical block, and this object is sized and positioned to not overlap the edge toward the start of the second vertical block. This object provides clear identification when a dog's nose has crossed the finish side edge of the first block (Starting Line 1).

The second and third blocks in the line away from the start recording device are marked to show the starting zones. A strip of 2.5 cm (1 in) wide light-colored (e.g., white or off-white) tape (e.g., masking tape) is adhered to the middle of the top surface of the second block perpendicular to the

long axis of the block. This separates the first starting zone from the second starting zone. The junction between the second and third blocks separates the second starting zone from the third starting zone. A similar strip of tape is adhered to the middle of the top surface of the third block. This delineates the end of the third starting zone. Small pieces of tape are placed in the middle of the three starting zones and marked with a "1", "2", and "3" from the start side to the finish side respectively to remind personnel which starting zone is which.

1.5 Course Setup

The ST course setup provides the dog a clear visual indication of where they are supposed to run and minimizes lateral movement that would affect performance. Continuous (e.g., fencing) or intermittent (e.g., cones) barriers are positioned to create a 2 m wide running course. The barriers are wider (e.g., 3-4 m) near the start to limit hesitation to run into an enclosed space but narrow quickly to the 2 m distance to encourage straight running. The barriers end slightly before the finish setup and recording device to enable visualization of the finish.

1.6 Finish Setup

The ST finish setup enables the finish performance of the dog to be assessed. The ST finish setup is shown in **Figure 2 (C)**. 25 m is measured from Starting Line 3 (finish edge of first block) through the middle of the running course. A small object is placed on the ground to mark this distance. A standard concrete block is placed 1 m outside the running course on the side away from the finish recording device. This block is oriented horizontally with its long edge parallel to the running course and its finish side short edge in line with the object placed at the 25 m distance. If this block has holes through it, the solid side is oriented towards the finish recording device. This block is assessed with a level to ensure it is level, primarily in the axis parallel to the running course. An additional block is oriented in a similar manner and stacked horizontally on top of the first block with all edges aligned.

Two additional blocks are stacked on top of these blocks with their long edges together (oriented vertically). These blocks form the three finish lines. Finish Line 1 is formed by the finish side edge of the finish side block, Finish Line 2 by the junction between the two blocks, and Finish Line 3 by the start side edge of the start side block. If the blocks have holes through them, the solid sides are oriented towards the finish recording device. As most blocks are slightly (e.g., 0.25 in or 0.5 cm) shorter in height than half of their length, the two vertical blocks are centered (in the axis parallel to the running course) on the horizontal block below them. The vertical edges of these blocks are assessed with a level to ensure they are vertical, primarily in the axis parallel to the running course.

A strip of 2.5 cm (1 in) wide light-colored (e.g., white or off-white) tape (e.g., masking tape) is adhered to the start side long edges of the two vertical blocks. The two tape strips are continued onto both horizontal blocks below. These strips provide clear identification when a dog's nose has crossed the start side edges of the start side and finish side blocks (Finish Line 3 and Finish Line 2 respectively). A high-contrast (e.g., white or off-white) object is placed behind the finish side edge of the finish side vertical block, and this object is sized and positioned to not overlap the start side edge of the start side vertical block. This object provides clear identification when the dog's nose has crossed the finish side edge of the finish side block (Finish Line 1).

1.7 Start and Finish Recording Device Setup

Two devices (e.g., video camera, camcorder, smartphone, or tablet) are used to capture the start and finish of the ST. The devices must record at least 60 frames per second at a resolution (e.g., 1280 x 1080 pixels) sufficient to assess the dog's nose movement from 5-7 m away. The recording format must be compatible with the device and software used for analysis (e.g., MP4 for analysis with Kinovea on a PC). The devices must have sufficient battery capacity (approximately 5-15 minutes per dog) and data storage capacity to record all expected dogs. This study used two handheld video cameras (HC-V180K Full HD Camcorder, Panasonic, Japan at 60 frames per second and 1280 x 1080 pixels) typically used for recording training activities at the PVWDC.

The devices are synchronized prior to the first attempt. The synchronization process supplies a common reference point on both devices for later analysis. Both devices are oriented towards a bright light source, video recording is started on both devices, and the light source is turned on. This study used a bright LED flashlight (Fury, SureFire, CA, USA) for ease of visualization outdoors. Both devices record continuously from the sync through the dog's attempts, and the recording on both devices is stopped at the conclusion of the last attempt.

The devices are placed at the start and finish locations. A tripod or similar object is used to position the devices 30-40 cm (12-16 in) off the ground. The behavior of the dog running near the devices must be considered, and this study used a concrete block oriented vertically as a stable platform for the video cameras. The start device is positioned in line with the Starting Line 2 and 5-7 m away. When positioning this device, the location of personnel at the start and the prior training of the dogs must be considered. As this study used working dogs primarily trained to heel on the handler's left side, the start device was positioned to the left of the start setup (from the perspective of the dog) so the handler could be positioned on the side away from the device to not block the view of the starting lines. The start device is oriented to just capture the last (towards the start edge) block in the start setup and therefore capture as much of the running course as possible. The finish device is positioned in line with Finish Line 2 and 5-7 m away. This device is oriented to center on the finish setup.

1.8 Reward Setup

A highly motivating reward is used to incentivize the dog to perform maximally. For many dogs, this reward will be a toy, although some may perform better for food, verbal reward, physical affection, or a simulated apprehension (bite sleeve or suit). The reward must be easy to get back from the dog to allow for physical and behavioral recovery between attempts. The reward is manipulated to help optimal positioning, a smooth start, and maximal effort throughout the attempt. For many dogs this is performed by the rewarder standing near the finish lines. The reward is shown to the dog to increase their arousal before they enter the start setup. The reward is then concealed, and the dog is positioned between the start blocks and into a down position. A similar or lower value reward (e.g., food) may be used to perform these tasks. When the person at the start signals the dog's readiness, the rewarder reveals the reward, calls the dog, and runs past the finish lines. For maximal performance and to prevent premature slowing, the rewarder must be at least 10 m beyond the finish lines when the dog reaches them. Using a toy thrown past the finish lines is not recommended as many dogs pause or slow their movement to track the flight of the toy. Once the dog reaches the rewarder, the rewarder supplies 10-15 seconds of highly engaging rewarding. The reward is then retrieved from the dog and concealed to enable physical and behavioral recovery before the next attempt.

2 Performance Protocol

Prior to performing the ST, data should be recorded for the dog. Items to record include the dog's name, the type of reward and how it will be manipulated, the location and surface type, the temperature, and the time of day. Unless otherwise needed and for maximum performance, the dog should not wear unnecessary equipment (e.g., harness). Any equipment worn beyond basic collars should be recorded.

To reduce the risk of injury and maximize performance, the dog must perform a warm-up. The dog should first perform a general warm-up (e.g., the PVWDC Fit to Work Warm-up) with an emphasis on off-leash walking and trotting and spinal, hip, stifle, and tarsal flexion and extension in a dynamic format. The dog should then perform a specific warm-up of off leash running and short (5-10 m) sprints from a down position.

Each attempt starts with the dog positioned between the lines of blocks at the start and oriented towards the finish lines. The dog must be in a complete (sternum, elbows, and hocks contacting the ground) square down position (e.g., the PVWDC Fit to Work Posture Down rather than a settle with the hindlimbs rotated to one side). A sit may be used if the dog has not been trained to perform a down position or is unable to perform a down position due to arousal. If a sit is used, this information should be recorded as this modified position will affect performance. The dog should be lightly restrained to prevent premature movement, especially when the rewarder begins moving. One or two fingers should be inserted under the caudal side of the dorsal portion of the collar to allow the dog to smoothly break free when directed.

The person at the start must ensure the most cranial aspect of the dog's chest (e.g., manubrium of sternum) is positioned in one of the starting zones. This zone then dictates the starting line and finish line used for analyzing the attempt. If the dog's chest is on the line between two zones, the person should try to manipulate the dog forward or backward slightly. If this is not possible due to the dog's temperament and arousal, the dog is in the rearward zone for measurement purposes (e.g., zone 2 if the dog's chest is on the line between zones 1 and 2). Once the dog is positioned appropriately, the person indicates the dog's readiness to the rewarder. This indication is typically done with a raised hand (vs. verbally) to limit further arousal of the dog. The starting zone is observed during the starting process but not recorded until after the attempt starts as the dog may move slightly (typically cranially) immediately prior to the start. The starting zone can be recorded manually, spoken verbally for the device to capture, or shown with fingers held where the device can capture.

The attempt is observed by the person at the start and by another observer to the side (if available). Two aspects of the attempt are observed, scored, and recorded. The first is the perceived effort and motivation of the dog. The purpose of this assessment is to add qualitative context to the quantitative attempt result. This is observed primarily by the person to the side and secondarily by the person at the start. The perceived effort ratings and descriptions are summarized in **Table 1**.

TABLE 1. ST perceived effort ratings and descriptions.

Perceived Effort Rating	Description
A	Highly motivated and running with earnest effort. Eager prior to the start, rises quickly from the ground, and runs all the way to the rewarder.

B	Slight decrease in effort (e.g., slight hesitation at the start).
C	Significant decrease in effort (e.g., visibly uninterested at the start but still performs the attempt).
F	Did not complete the attempt (e.g., leaves the running course to investigate something else).

The test quality is also observed, scored, and recorded. The purpose of this assessment is to document any effect of a test element on the dog's performance. The test quality ratings and descriptions are summarized in **Table 2**.

TABLE 2. ST test quality ratings and descriptions.

Test Quality Rating	Description
A	Perfectly executed attempt with no performance degrading factors.
B	Slight impact on the dog's performance (e.g., rewarder too close to the finish lines, causing the dog to slow prematurely).
C	Significant impact on the dog's performance (e.g., inappropriate restraint at the start resulting in a rough start).
F	One or more test elements that prevents the attempt from being performed (e.g., a nearby animal causing the dog to be unable to focus on the attempt).

After completion of the first attempt and retrieving the reward, the dog is allowed at least 90 seconds of rest to recover. Efforts must be made to ensure this period allows for full recovery, especially regarding physical activity (standing or walking are preferred) thermal stress (shade or cooling pool if needed) and environmental stimulation (minimal interaction with people or other dogs).

To accurately assess the dog's performance, the goal is for them to complete three attempts where they both receive an "A" for effort and motivation, and the test quality is an "A". To limit the effect of fatigue, the dog is allowed up to five attempts to accumulate these three desired attempts. In the case of test quality issues, the dog may be allowed more attempts.

After completing all necessary attempts, the dog must be properly cooled down. The dog must be assessed for thermal stress and proper therapeutic measures applied. If the dog is continuing to another physical assessment, the physical part of the cool-down may solely consist of on-leash walking. If the dog is returning to rest, they should perform a thorough cool-down (e.g., the PVWDC Fit to Work Cool-down) with an emphasis on on-leash walking and spinal, hip, stifle, and tarsal flexion and extension in a static format.

3 Measurement Protocol

After recording the dog's ST attempts, the start and finish video files are analyzed to determine the dog's quantitative performance. If a standalone video recording device (e.g., video camera) is used,

the files are transferred to a device for analysis. If a smartphone, tablet, or similar device is used, the files may be analyzed on that device. Software suitable for frame-by-frame analysis and magnification of portions of the video is used. This study used Kinovea (version 0.8.27), a free 2D motion analysis software running on desktop computers.

The start and finish videos are synchronized using the dual screen mode. Each video is analyzed to find the first frame where the flashlight turned on. The elapsed time on the video (to the millisecond) is identified and recorded. An on-screen timer is added to each video and used to aid in finding the approximate finish time of each attempt.

The start video is analyzed to find the start time of the first attempt. The area around the starting lines is magnified 2.5x to aid in finding the start time. The approximate start time of the first attempt is found by reviewing the video at high speed (e.g., 2-3x) and then slowing when the dog entered the start setup. The start video is advanced past the actual start of the first attempt until the starting zone of the first attempt is shown in the video by the person at the start. The video is then reversed frame by frame until the first frame when the dog's nose crosses the corresponding starting line. The elapsed time on the video (to the millisecond) is identified as the start time and recorded. The start video is then evaluated at 0.25x speed from the time the dog begins moving until they leave the screen to assess their movement. Any abnormalities in the dog's movement (e.g., unequal foot placement or decreased joint range of motion) and any previously unrecognized quality issues (e.g., not fully in a down position or excessive restraint) are recorded.

The finish video is advanced to 2.5 seconds after the first attempt start time (using the on-screen timer) and reviewed at slow speed (e.g., 0.25-0.5x) until the dog crosses the finish lines. The video is then reversed frame by frame until the first frame when the dog's nose crosses the corresponding finish line. The elapsed time on the video (to the millisecond) is identified as the finish time and recorded.

This process is repeated for all attempts. The duration of each attempt is calculated by adjusting the start time (Ts) and finish (Tf) times by the synchronized time (STs and STf) and then subtracting the adjusted start time from the adjusted finish time (e.g., (Tf-STf) - (Ts-STs)). The shortest duration from a properly performed attempt ("A" for both perceived effort and test quality) is then reported as that dog's result.
